# Increased risk of uveitis and optic neuritis after herpes zoster reactivation in COVID-19: a TriNetX database study

**DOI:** 10.1093/qjmed/hcaf332

**Published:** 2026-01-06

**Authors:** Yi-Hsin Lin, Man-Sze Wong, Shan-Shy Wen, Ai-Ling Hour, Chien-Lin Lu, Ming Ling Tsai, Kuo-Cheng Lu, Yu-Chen Cheng

**Affiliations:** School of Medicine, College of Medicine, Fu Jen Catholic University, New Taipei City, Taiwan; Department of Medical Education, National Taiwan University Hospital, Taipei, Taiwan; Graduate Institute of Applied Sciences and Engineering, College of Sciences and Engineering, Fu Jen Catholic University, New Taipei City, Taiwan; Department of Life Science, College of Sciences and Engineering, Fu Jen Catholic University, New Taipei City, Taiwan; School of Medicine, College of Medicine, Fu Jen Catholic University, New Taipei City, Taiwan; Division of Nephrology, Department of Internal Medicine, Fu Jen Catholic University Hospital, School of Medicine, College of Medicine, Fu Jen Catholic University, New Taipei City, Taiwan; Department of Ophthalmology, Taipei Buddhist Tzu Chi Hospital, Buddhist Tzu Chi Medical Foundation, New Taipei City, Taiwan; Division of Nephrology, Department of Internal Medicine, Fu Jen Catholic University Hospital, School of Medicine, College of Medicine, Fu Jen Catholic University, New Taipei City, Taiwan; Division of Nephrology, Department of Medicine, Taipei Tzu Chi Hospital, Buddhist Tzu Chi Medical Foundation, New Taipei City, Taiwan; School of Medicine, College of Medicine, Fu Jen Catholic University, New Taipei City, Taiwan; Department of Neurology, Fu Jen Catholic University Hospital, Fu Jen Catholic University, New Taipei City, Taiwan

## Abstract

**Background:**

Herpes zoster (HZ) reactivation following COVID-19 suggests immune dysregulation, posing a risk for long-term neuro-ophthalmic sequelae.

**Aim:**

This study investigates the relationship between post-COVID HZ and the subsequent hazard of uveitis and optic neuritis.

**Design:**

This retrospective analysis used the TriNetX Research Network, following patients for 3 years.

**Methods:**

Propensity score matching (PSM) was performed on COVID-19 survivors, comparing the HZ-exposed group (*n* = 11 172) against a matched HZ-unexposed control group (*n* = 11 173). Cox proportional hazards models calculated the 3-year Hazard Ratios (HRs) for incident uveitis and optic neuritis

**Results:**

Post-COVID−HZ reactivation was associated with a significantly elevated and persistent risk of both outcomes. HZ was consistently related to a 3.06-fold increase in the hazard of uveitis (HR = 3.06; 95% CI, 2.56–3.65; *P* < 0.001) and a 2.10-fold increase in the hazard of optic neuritis (HR = 2.10; 95% CI, 1.62–2.71; *P* < 0.001). The relationships remained significant across sensitivity analyses, and the risk remained high even in the vaccinated subgroup.

**Conclusions:**

HZ reactivation after COVID-19 is related to a higher instantaneous risk of subsequent uveitis and optic neuritis, even among immunocompetent individuals. HZ may serve as a clinical marker for neuro-immune injury, supporting the consideration of targeted ophthalmic surveillance and timely antiviral intervention to prevent vision loss.

## Introduction

Herpes zoster ophthalmicus (HZO) results from the reactivation of latent varicella-zoster virus (VZV) within the ophthalmic division of the trigeminal nerve. Accounting for approximately 10–20% of all herpes zoster (HZ) cases,^1^ HZO poses a significant risk of long-term ocular morbidity.^2^^,^^3^ Ocular manifestations, particularly anterior uveitis and optic neuritis, carry a high risk of permanent visual morbidity.^4^^,^^5^ These complications—driven by direct viral invasion or post-infectious immune mechanisms—can progress to secondary glaucoma and irreversible severe vision loss if not promptly managed.^6^^,^^7^

The global SARS-CoV-2 pandemic introduced a new layer of complexity to post-infectious diseases. COVID-19 infection has been unequivocally linked to a variety of post-acute inflammatory sequelae, including secondary ocular and neuro-ophthalmic inflammation such as uveitis and optic neuritis. This susceptibility is attributed to virus-induced immune dysregulation, endothelial injury, and post-viral autoimmune activation.^8–11^

Crucially, an increasing number of clinical observations and large cohort studies suggest that COVID-19 may significantly increase the risk of HZ reactivation.^12–14^ This is primarily attributed to virus-induced immune dysregulation, transient lymphopenia, and impaired T-cell surveillance of latent VZV. The co-occurrence of these two factors—SARS-CoV-2 infection leading to immune shifts and VZV reactivation leading to neuro-ophthalmic inflammation—raises significant concern for overlapping pathogenic pathways that could synergistically enhance the risk of uveitis and optic neuritis in COVID-19 survivors.

However, current evidence exploring this potential synergy is limited by small sample sizes, retrospective case observations, and a general lack of appropriately matched control groups. To date, no large-scale, multi-institutional study has rigorously evaluated whether a history of COVID-19 infection may modify or exacerbate the risk of HZ-associated ocular inflammation. To address this critical knowledge gap, we conducted a rigorous retrospective database study using the TriNetX Research Network. The primary objective of this study was to assess whether post-COVID-19 HZ reactivation is associated with a significantly increased long-term risk of uveitis and optic neuritis when compared to HZ reactivation in non-COVID-19 individuals.

## Materials and methods

### Study design and data source

This research was a retrospective, longitudinal database study utilizing the TriNetX Global Collaborative Network, a global federated health research network comprising de-identified electronic health records from 144 international healthcare organizations. The data provided structured information including demographics, diagnostic codes, procedures, prescribed medications, and laboratory results. All statistical computations were performed on the TriNetX platform’s secure analysis module on 10 October 2025. The study adhered to the ethical principles of the Declaration of Helsinki. Ethics approval was obtained from the Institutional Review Board (IRB) of Taipei Tzu Chi Hospital (Approval No.: 14-IRB140), with a waiver of informed consent granted.

### Study population

The study focused on adult patients (age ≥ 18 years) with a confirmed COVID-19 diagnosis (International Classification of Diseases codes (ICD)-10-CM U07.1 or positive SARS-CoV-2 lab result) recorded between 1 January 2020 and 31 December 2022. The index date was defined as the first confirmed COVID-19 diagnosis. Patients with a documented history of HZ (ICD-10 B02, B02.X) within 1 year preceding the index date were excluded to ensure temporality.

**Figure 1.**
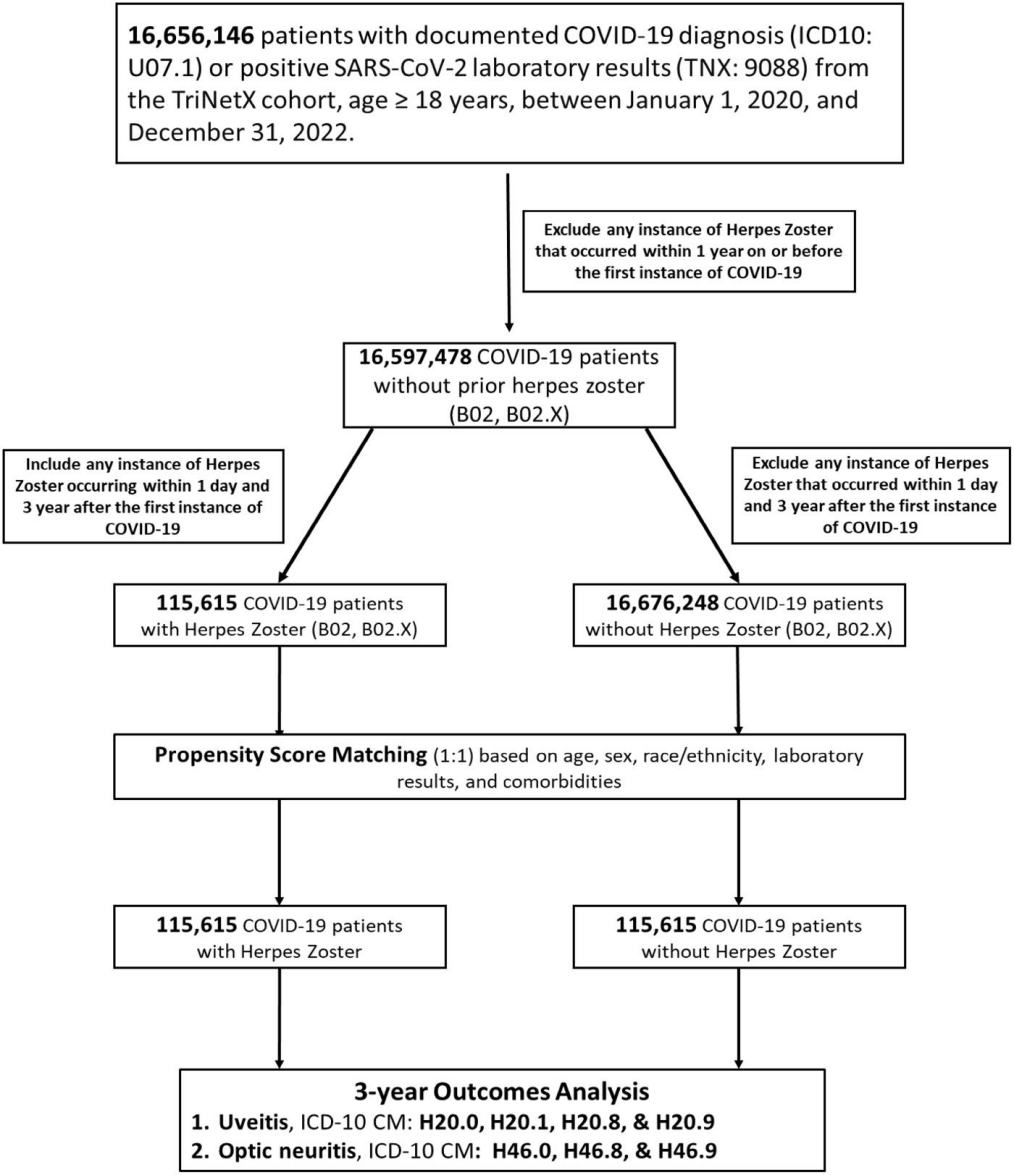
Study population assembly and PSM analytic workflow. This schematic outlines the sequential methodology utilized for generating the study groups from an initial population of 16 656 146 adult survivors of COVID-19. Individuals with a previous diagnosis of HZ occurring before or within 1 year of the COVID-19 index date were systematically filtered out. The determination of group exposure was based on the diagnosis of HZ within the 3-year post-COVID follow-up window (defined as 1 day to 3 years post-index). This process established two principal groups: the HZ-exposed group (*n* = 115 615), which includes patients who developed HZ in the specified period, and the HZ-unexposed control group (*n* = 16 676 248), comprising patients who remained free of an HZ diagnosis throughout the follow-up. Subsequent to this identification, a 1:1 PSM procedure was applied to balance the groups, resulting in two final groups of 115 615 patients each used for comparative risk analysis of uveitis and optic neuritis.

The remaining patients were divided into two study groups for the post-COVID period (1 day to 2 years post-index): the Exposed Group (COVID-19+HZ), including patients with a new HZ diagnosis, and the unexposed group (COVID-19−HZ), comprising individuals without an HZ diagnosis. The initial source population comprised 115 675 exposed and 16 676 248 unexposed patients (Figure 1).

### Propensity score matching and outcome measures

Due to platform constraints with the extensive source population (over 16 million individuals), a primary 1:1 propensity score matching (PSM) was implemented using seven key variables (age, sex, race/ethnicity, diabetes mellitus, and hypertension), reflecting the maximum allowable set for a study group of this magnitude. PSM utilized a greedy nearest-neighbor algorithm with a caliper width of 0.1.

To address potential unmeasured confounding, we performed an expanded validation analysis in the 50–59 age stratum using 29 clinical and laboratory variables, including inflammatory markers and medication history (Table 1, Supplementary Table S2). Group balance was confirmed by standardized mean differences (SMD < 0.1) and visualized via love plots (Supplementary Figure S1). Primary outcomes included incident uveitis (ICD-10 H20.0, H20.1, H20.8, and H20.9) and optic neuritis (ICD-10 H46.0, H46.8, and H46.9) during a 3-year (1095 days) follow-up, excluding patients with pre-existing conditions. Diagnostic validity was reinforced by requiring specialist confirmation and recurrent ICD-10 coding, while healthcare utilization was analyzed to mitigate potential detection bias.

**Table 1:** Baseline characteristics of COVID-19 patients aged 50–59 years with vs. without subsequent herpes zoster reactivation, before and after propensity score matching

Characteristics	Before matching				After matching			
	Mean ± SD	Patient count	% of group	Std. diff.	Mean ± SD	Patient count	% of group	Std. diff.
Demographics								
Age at Index	54.49 ± 3.00 vs. 54.39 ± 2.95	22 345 vs. 2 583 470	100.00% vs. 100.00%	0.033	54.49 ± 3.00 vs. 54.45 ± 2.99	22 345 vs. 22 345	100.00% vs. 100.00%	0.013
Current age	58.92 ± 3.18 vs. 58.80 ± 3.10	22 345 vs. 2 583 470	100.00% vs. 100.00%	0.038	58.92 ± 3.18 vs. 58.87 ± 3.16	22 345 vs. 22 345	100.00% vs. 100.00%	0.014
Female		14 785 vs. 1 359 632	66.17% vs. 52.63%	0.278		14 785 vs. 14 607	66.17% vs. 65.37%	0.017
Male		7556 vs. 1 220 726	33.81% vs. 47.25%	0.276		7556 vs. 7 723	33.81% vs. 34.56%	0.016
White		15 659 vs. 1 440 352	70.08% vs. 55.75%	0.300		15 659 vs. 15 858	70.08% vs. 70.97%	0.020
Black or African American		2584 vs. 366 662	11.56% vs. 14.19%	0.079		2584 vs. 2 641	11.56% vs. 11.82%	0.008
Unknown ethnicity		4659 vs. 843 255	20.85% vs. 32.64%	0.269		4659 vs. 4 536	20.85% vs. 20.30%	0.014
Hispanic or Latino		1731 vs. 185 516	7.75% vs. 7.18%	0.022		1731 vs. 1 699	7.75% vs. 7.60%	0.005
Not Hispanic or Latino		15 955 vs. 1 554 699	71.40% vs. 60.18%	0.238		15 955 vs. 16 110	71.40% vs. 72.10%	0.015
Asian		1044 vs. 102 467	4.67% vs. 3.97%	0.035		1044 vs. 923	4.67% vs. 4.13%	0.026
Diagnosis								
Hypertensive diseases		7431 vs. 499 702	33.26% vs. 19.34%	0.320		7431 vs. 7 509	33.26% vs. 33.60%	0.007
Diabetes mellitus		3675 vs. 237 808	16.45% vs. 9.21%	0.218		3675 vs. 3 657	16.45% vs. 16.37%	0.002
Ischemic heart diseases		1420 vs. 101 456	6.36% vs. 3.93%	0.110		1420 vs. 1 376	6.36% vs. 6.16%	0.008
Cerebrovascular diseases		567 vs. 43 611	2.54% vs. 1.69%	0.059		567 vs. 572	2.54% vs. 2.56%	0.001
Medications								
Antilipemic agents		4595 vs. 320 659	20.56% vs. 12.41%	0.221		4595 vs. 4 585	20.56% vs. 20.52%	0.001
Blood glucose regulation agents		3743 vs. 247 141	16.75% vs. 9.57%	0.214		3743 vs. 3 675	16.75% vs. 16.45%	0.008
Diuretics		3476 vs. 227 917	15.56% vs. 8.82%	0.207		3476 vs. 3 507	15.56% vs. 15.70%	0.004
Beta-blockers		3161 vs. 217 446	14.15% vs. 8.42%	0.182		3161 vs. 3 080	14.15% vs. 13.78%	0.010
Calcium channel blockers		2421 vs. 172 372	10.84% vs. 6.67%	0.148		2421 vs. 2 392	10.84% vs. 10.71%	0.004
ACE inhibitors		2315 vs. 171 073	10.36% vs. 6.62%	0.134		2315 vs. 2 302	10.36% vs. 10.30%	0.002
Angiotensin II inhibitor		2002 vs. 134 023	8.96% vs. 5.19%	0.148		2002 vs. 1 952	8.96% vs. 8.74%	0.008
Laboratory results								
Creatinine	1.06 ± 3.25 vs. 1.06 ± 2.94	12 603 vs. 884 597	56.40% vs. 34.24%	0.001	1.06 ± 3.25 vs. 1.04 ± 3.02	12 603 vs. 12 765	56.40% vs. 57.13%	0.008
Sodium	139.07 ± 2.94 vs. 139.03 ± 3.02	12 593 vs. 892 668	56.36% vs. 34.55%	0.015	139.07 ± 2.94 vs. 139.04 ± 2.92	12 593 vs. 12 853	56.36% vs. 57.52%	0.011
Potassium	4.17 ± 0.45 vs. 4.16 ± 0.47	12 456 vs. 879 245	55.74% vs. 34.03%	0.011	4.17 ± 0.45 vs. 4.16 ± 0.47	12 456 vs. 12 713	55.74% vs. 56.89%	0.017
Glucose	116.83 ± 54.64 vs. 116.39 ± 52.97	12 371 vs. 875 656	55.36% vs. 33.90%	0.008	116.83 ± 54.64 vs. 117.16 ± 54.12	12 371 vs. 12 622	55.36% vs. 56.49%	0.006
Calcium	9.36 ± 0.56 vs. 9.34 ± 0.59	12 246 vs. 835 776	54.80% vs. 32.35%	0.034	9.36 ± 0.56 vs. 9.35 ± 0.56	12 246 vs. 12 379	54.80% vs. 55.40%	0.015
Urea nitrogen	16.27 ± 9.10 vs. 16.10 ± 9.01	11 987 vs. 800 478	53.65% vs. 30.98%	0.018	16.27 ± 9.10 vs. 16.10 ± 9.09	11 987 vs. 12 092	53.65% vs. 54.12%	0.018
Hemoglobin	13.47 ± 1.84 vs. 13.58 ± 1.93	11 574 vs. 818 453	51.80% vs. 31.68%	0.055	13.47 ± 1.84 vs. 13.48 ± 1.89	11 574 vs. 11 726	51.80% vs. 52.48%	0.006
Platelets	257.11 ± 81.01 vs. 255.95 ± 81.82	11 471 vs. 810 799	51.34% vs. 31.38%	0.014	257.11 ± 81.01 vs. 260.31 ± 81.69	11 471 vs. 11 630	51.34% vs. 52.05%	0.039
Alanine aminotransferase	29.05 ± 134.78 vs. 29.80 ± 53.15	11 371 vs. 770 952	50.89% vs. 29.84%	0.007	29.05 ± 134.78 vs. 29.39 ± 46.53	11 371 vs. 11 517	50.89% vs. 51.54%	0.003
Aspartate aminotransferase	26.46 ± 35.45 vs. 29.10 ± 77.76	11 194 vs. 749 833	50.10% vs. 29.02%	0.044	26.46 ± 35.45 vs. 29.02 ± 83.48	11 194 vs. 11 326	50.10% vs. 50.69%	0.040
Albumin	4.14 ± 0.46 vs. 4.13 ± 0.51	10 928 vs. 726 343	48.91% vs. 28.11%	0.035	4.14 ± 0.46 vs. 4.13 ± 0.50	10 928 vs. 11 035	48.91% vs. 49.38%	0.028
Alkaline phosphatase	87.42 ± 51.96 vs. 87.70 ± 57.30	10 875 vs. 722 348	48.67% vs. 27.96%	0.005	87.42 ± 51.96 vs. 87.50 ± 55.39	10 875 vs. 10 973	48.67% vs. 49.11%	0.001
Bilirubin. total	0.56 ± 0.63 vs. 0.62 ± 0.98	10 699 vs. 710 157	47.88% vs. 27.49%	0.070	0.56 ± 0.63 vs. 0.60 ± 0.85	10 699 vs. 10 807	47.88% vs. 48.36%	0.051
Leukocytes	49.31 ± 417.87 vs. 25.11 ± 273.33	10 387 vs. 722 819	46.48% vs. 27.98%	0.069	49.31 ± 417.87 vs. 26.54 ± 284.26	10 387 vs. 10 498	46.48% vs. 46.98%	0.064
Protein	7.10 ± 0.64 vs. 7.13 ± 0.75	10 248 vs. 689 185	45.86% vs. 26.68%	0.042	7.10 ± 0.64 vs. 7.11 ± 0.82	10 248 vs. 10 377	45.86% vs. 46.44%	0.014
Cholesterol in HDL	53.17 ± 17.48 vs. 52.09 ± 17.28	8053 vs. 505 021	36.04% vs. 19.55%	0.062	53.17 ± 17.48 vs. 52.96 ± 17.77	8053 vs. 8058	36.04% vs. 36.06%	0.012
Cholesterol. total	192.20 ± 45.86 vs. 189.17 ± 44.71	8037 vs. 497 276	35.97% vs. 19.25%	0.067	192.20 ± 45.86 vs. 190.99 ± 44.64	8037 vs. 8027	35.97% vs. 35.92%	0.027
Cholesterol in LDL	111.21 ± 38.89 vs. 109.92 ± 37.53	7988 vs. 498 572	35.75% vs. 19.30%	0.034	111.21 ± 38.89 vs. 111.02 ± 37.83	7988 vs. 8016	35.75% vs. 35.87%	0.005
Triglyceride	146.22 ± 118.54 vs. 144.16 ± 127.44	7959 vs. 503 126	35.62% vs. 19.48%	0.017	146.22 ± 118.54 vs. 147.14 ± 280.93	7959 vs. 8018	35.62% vs. 35.88%	0.004
Hemoglobin A1c	6.55 ± 1.76 vs. 6.47 ± 1.70	5942 vs. 387 176	26.59% vs. 14.99%	0.044	6.55 ± 1.76 vs. 6.50 ± 1.71	5942 vs. 5909	26.59% vs. 26.44%	0.025
C reactive protein	16.78 ± 38.89 vs. 23.67 ± 49.43	1943 vs. 120 373	8.70% vs. 4.66%	0.155	16.78 ± 38.89 vs. 18.95 ± 45.58	1943 vs. 1914	8.70% vs. 8.57%	0.051
0–10 mg/l		1469 vs. 81 496	6.57% vs. 3.15%	0.159		1469 vs. 1403	6.57% vs. 6.28%	0.012
10–30 mg/l		463 vs. 30 381	2.07% vs. 1.18%	0.071		463 vs. 465	2.07% vs. 2.08%	0.001
Calcidiol	34.99 ± 16.85 vs. 33.96 ± 16.95	1745 vs. 89 106	7.81% vs. 3.45%	0.061	34.99 ± 16.85 vs. 34.61 ± 17.03	1745 vs. 1658	7.81% vs. 7.42%	0.022
Ferritin (ng/ml)	296.74 ± 859.17 vs. 273.22 ± 830.93	1652 vs. 100 132	7.39% vs. 3.88%	0.028	296.74 ± 859.17 vs. 267.89 ± 1459.36	1652 vs. 1570	7.39% vs. 7.03%	0.024
0–100		899 vs. 53 338	4.02% vs. 2.06%	0.114		899 vs. 886	4.02% vs. 3.96%	0.003
100–200		360 vs. 22 678	1.61% vs. 0.88%	0.066		360 vs. 367	1.61% vs. 1.64%	0.002
200–300		171 vs. 11 017	0.77% vs. 0.43%	0.044		171 vs. 166	0.77% vs. 0.74%	0.003
300–600		226 vs. 12 469	1.01% vs. 0.48%	0.061		226 vs. 172	1.01% vs. 0.77%	0.026
600–1000		107 vs. 6 186	0.48% vs. 0.24%	0.040		107 vs. 104	0.48% vs. 0.47%	0.002
Iron	73.74 ± 41.05 vs. 74.85 ± 43.88	1546 vs. 91 035	6.92% vs. 3.52%	0.026	73.74 ± 41.05 vs. 73.27 ± 42.46	1546 vs. 1480	6.92% vs. 6.62%	0.011

COVID-19, coronavirus disease 2019; St Diff, standardized difference; values <0.1 typically indicate good balance.

### Statistical analysis

Continuous variables were summarized as the mean ± SD, and categorical variables as frequencies and percentages (%). Baseline differences between the exposed and unexposed patient groups were assessed using Student’s *t*-test for continuous variables and the chi-squared test for categorical variables.

Crude incidence was described using ORs, while primary time-to-event analyses utilized Kaplan–Meier curves and Cox proportional hazards models to estimate HRs. To address potential survival bias, we performed a competing risk analysis using the Aalen–Johansen estimator, where all-cause mortality was treated as a competing event. The proportional hazards assumption was verified via scaled Schoenfeld residuals (*P* > 0.05).

### Sensitivity analysis

To ensure the reliability of the findings, we implemented an extensive sensitivity analysis framework. First, we conducted an age-restricted validation analysis (50–59 years) using 29 covariates to confirm the primary PSM model; furthermore, subgroup analyses were performed across various clinical strata and visualized via forest plots to ensure consistency across populations. Second, temporal stability was evaluated through landmark analyses (at 1, 2, and 3 years) and time-window–based sensitivity analysis (HZ onset within 6, 12, 24, or 36 months) to account for exposure timing. Third, we reinforced diagnostic validity by requiring ophthalmology specialist confirmation and recurrent coding, while analyzing hospitalization burden and healthcare utilization to account for differences in clinical severity and potential detection bias.

Finally, we mitigated clinical confounding by excluding individuals with HIV or prior immunosuppression, comparing cumulative COVID-19 reinfection counts, and stratifying results by vaccination and antiviral treatment status. All tests were two-sided (*P* < 0.05), with analyses primarily performed within TriNetX and additional computations for exploratory vaccination subgroups performed manually using IBM SPSS Statistics, Version 20.0.

## Results

### Group characteristics and primary findings

The analysis included 22 345 well-matched patients (*n* = 11 172 in the HZ-exposed and *n* = 11 173 in the HZ-unexposed groups). Following PSM, both groups achieved excellent covariate balance (all SMDs <0.1), effectively mitigating confounding bias (Table 1). COVID-19 survivors with HZ reactivation demonstrate a significantly higher instantaneous risk of post-infectious ocular inflammatory complications compared with non-reactivated individuals over the 3-year follow-up.

### Uveitis

The HZ-exposed group exhibited a significantly higher incidence of uveitis compared to the unexposed group. The crude incidence analysis showed HZ exposure was associated with nearly four times the odds of developing uveitis (OR = 3.934; 95% CI, 3.297–4.694; *P* < 0.001) compared to the non-exposed group (Supplementary Table S1).

**Figure 2.**
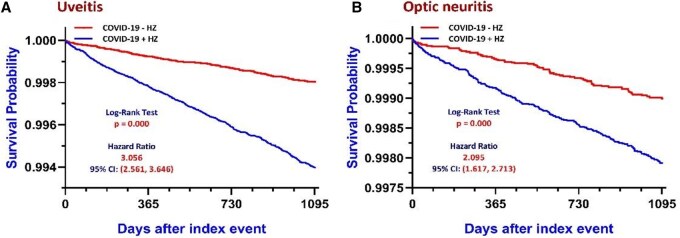
Kaplan–Meier survival curves comparing COVID-19 patients with and without HZ reactivation. These curves depict the cumulative incidence of freedom from (A) uveitis and (B) optic neuritis over the 3-year follow-up period. The data compares PSM groups of COVID-19 survivors who experienced HZ reactivation (blue line) vs. those who did not (red line). The persistent separation of the curves indicates a sustained, significantly elevated risk associated with HZ reactivation. Statistical significance for the separation between the survival distributions was determined using the log-rank test, with the resulting *P*-values detailed within each panel.

**Figure 3.**
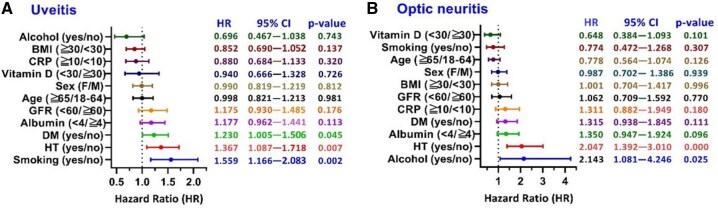
Subgroup analysis of hazard ratios for uveitis and optic neuritis following HZ reactivation. This figure presents the results of the subgroup analysis for the 3-year risk of incident neuro-ophthalmic outcomes: (A) uveitis and (B) optic neuritis, comparing HZ-exposed vs. HZ-unexposed COVID-19 survivors. The analysis is stratified by baseline factors, including demographics, cardiometabolic conditions (such as hypertension and diabetes), and behavioral factors (such as smoking and alcohol use). Each row in the plot shows the HR and 95% CI for the relationship within that specific subgroup. The vertical line at HR = 1.0 indicates no difference in risk between the HZ-exposed and unexposed groups. The results highlight potential patterns of effect modification by key systemic factors across the two outcomes. DM, diabetes mellitus; HT, hypertension.

Cox proportional hazards modeling confirmed that HZ reactivation significantly increased the instantaneous risk of uveitis (HR = 3.056; 95% CI, 2.561–3.646; *P* < 0.001) (Figure 2A). Subgroup analysis (Figure 3A) identified several risk factors for incident uveitis: diabetes mellitus (HR = 1.230; 95% CI, 1.005–1.506), hypertension (HR = 1.367; 95% CI, 1.087–1.718), and smoking (HR = 1.559; 95% CI, 1.166–2.083).

### Optic neuritis

Patients with HZ reactivation showed a significantly increased instantaneous risk of developing optic neuritis. The crude incidence analysis indicated odds were approximately 2.7 times higher in the HZ-exposed group (OR = 2.679; 95% CI, 2.069–3.470; *P* < 0.001) (Supplementary Table S1).

Cox proportional hazards modeling identified HZ reactivation as being related to a higher instantaneous risk of optic neuritis. (HR = 2.095; 95% CI, 1.617–2.713; *P* < 0.001) (Figure 2B). Subgroup analysis (Figure 3B) indicated that hypertension (HR = 2.047; 95% CI, 1.392–3.010; *P* < 0.001) and alcohol use (HR = 2.143; 95% CI, 1.081–4.246; *P* = 0.025) were related to a higher hazard of optic neuritis.

### Sensitivity analyses

The primary findings remained consistent across multiple sensitivity analyses. The relationship was consistent across the age-restricted group and the overall group (Supplementary Table S2) and persistent throughout 1-, 2-, and 3-year landmark intervals (Supplementary Table S3). Comparison of crude and PSM-adjusted hazards (Supplementary Table S4) confirmed the HRs remained highly significant after adjustment (uveitis: HR = 3.056; optic neuritis: HR = 2.095; all *P* < 0.001). Time-window–based analysis demonstrates that the instantaneous risk of uveitis and optic neuritis remained consistent regardless of whether HZ onset occurred within 6, 12, 24, or 36 months following COVID-19 (Supplementary Table S10). Statistical validity was supported by satisfied proportional hazards assumptions (Supplementary Table S11) and consistent results in competing risk models (Supplementary Table S12).

Crucially, excluding individuals with pre-existing immunosuppression did not substantially attenuate the instantaneous risk (uveitis: HR = 2.970; 95% CI, 2.393–3.687; optic neuritis: HR = 2.226, 95% CI, 1.620–3.059; all *P* < 0.001), indicating that the findings are not limited to immunocompromised populations (Supplementary Table S5). Also, when excluding patients with HIV infection, the increased risk remained significant for both uveitis (HR 2.734; 95% CI 2.306–3.242, *P* < 0.001) and optic neuritis (HR 2.095; 95% CI 1.617–2.714, *P* < 0.001). To ensure diagnostic validity, the relationship remained significant after restricting outcomes to those confirmed by ophthalmology specialists. Both groups averaged more than three ophthalmology-related visits (3.18 ± 3.76 vs. 3.02 ± 3.45) (Supplementary Table S7A) and recorded >1 ICD-10 entry per patient (Supplementary Table S7B). Finally, while the HZ-exposed group exhibited a higher hospitalization burden (1.85 ± 5.89 vs. 1.01 ± 3.58; *P* < 0.001) (Supplementary Table S8) and higher 3-year COVID-19 reinfection counts (2.59 ± 1.48 vs. 1.48 ± 1.25; *P* < 0.001) (Supplementary Table S9).

### Vaccination and antiviral treatment effects

Vaccination significantly reduced the background hazard in the unexposed (COVID−HZ) group (uveitis HR = 0.709; 95% CI, 0.664–0.757; *P* < 0.0001; optic neuritis HR = 0.878; 95% CI, 0.798–0.965; *P* = 0.007), no significant protective effect was observed within the exposed (COVID+HZ) group (Table 2). Individuals with both COVID-19 and HZ maintained elevated hazards of neuro-ophthalmic complications regardless of vaccination status (uveitis HR = 2.669; optic neuritis HR = 2.906; *P* < 0.0001 for both; Table 3). Additionally, anti-SARS-CoV-2 medications did not significantly modify the 3-year hazard of complications (0.712% vs. 0.603%; *P* = 0.684; Supplementary Table S6).

**Table 2:** Within-group analysis of the effect of COVID-19 vaccination on the hazard of uveitis and optic neuritis^a^

Outcomes	Cohort characteristics				KM—survival analysis			
	Cohort (comparison)	Patients in cohort	Patients with outcome	OR	Survival probability	HR	95% CI	Log-rank test *P* value
Uveitis	COVID-19 + HZ Vaccine (− vs.+)	18 543 vs. 18 485	101 vs. 122	0.824	99.406% vs. 99.315%	0.871	(0.669–1.134)	0.305
	COVID-19−HZ Vaccine (− vs.+)	1 116 942 vs. 1 114 006	1400 vs. 2541	0.549	99.817% vs. 99.744%	0.709	(0.664–0.757)	<0.0001
Optic neuritis	COVID-19+HZ Vaccine (− vs.+)	18 620 vs. 18 596	36 vs. 43	0.836	99.796% vs. 99.761%	0.874	(0.561–1.361)	0.550
	COVID-19−HZ Vaccine (− vs. +)	1 119 138 vs. 1 118 128	722 vs. 1054	0.684	99.907% vs. 99.895%	0.878	(0.798–0.965)	0.007

a This table presents the HRs and 95% CIs for the two neuro-ophthalmic outcomes  analyzing the effect of COVID-19 vaccination within two distinct groups (vaccinated vs. unvaccinated): the HZ-exposed group (COVID+HZ) and the HZ-unexposed group (COVID−HZ). This analysis specifically determines if vaccination provides a protective effect *regardless* of HZ exposure status. COVID+HZ, COVID-19 patients with herpes zoster reactivation; COVID−HZ, COVID-19 patients without herpes zoster reactivation; KM, Kaplan–Meier.

**Table 3:** Impact of HZ reactivation on neuro-ophthalmic outcomes: between-group comparison stratified by COVID-19 vaccination status^a^

Outcomes	Cohort characteristics				KM survival analysis			
	Cohort (comparison)	Patients in cohort	Patients with outcome	OR	Survival probability	HR	95% CI	**Log-rank test** ***P* value**
Uveitis	Vaccination C+H vs. C−H	18 485 vs. 18 555	122 vs. 43	2.860	99.315% vs. 99.746%	2.669	(1.885 –3.778)	<0.0001
	No vaccination C+H vs. C−H	90 686 vs. 90 990	483 vs. 124	3.924	99.415% vs. 99.80%	2.987	(2.452–3.639)	<0.0001
**Optic neuritis**	Vaccination C+H vs. C−H	18 596 vs. 18 639	43 vs. 14	3.083	99.761% vs. 99.919%	2.906	(1.59–5.312)	0.003
	No vaccination C+H vs. C−H	91 047 vs. 91 203	168 vs. 61	2.762	99.798% vs. 99.907%	2.126	(1.586–2.85)	<0.0001

a This table presents the HRs and 95% CIs for uveitis and optic neuritis, comparing the COVID+HZ group vs. the COVID−HZ group. The comparison is stratified by vaccination status (i.e. comparing vaccinated COVID+HZ vs. vaccinated COVID−HZ, and unvaccinated COVID+HZ vs. unvaccinated COVID−HZ). The results determine if the elevated risk conferred by HZ persists or is mitigated by prior COVID-19 vaccination. C+H: COVID-19 patients with herpes zoster reactivation; C−H: COVID-19 patients without herpes zoster reactivation; KM, Kaplan–Meier.

## Discussion

This large-scale, longitudinal evidence suggests that HZ reactivation represents a clinically meaningful post-infectious complication of COVID-19 with sustained neuro-ophthalmic consequences. The consistency of these findings across multiple sensitivity analytic strategies—including persistent Kaplan–Meier separation over 3 years—underscores the reliability of this relationship and minimizes the likelihood of residual confounding.

### Biological plausibility and mechanisms

The heightened risks have a clear biological basis, reflecting the synergistic effects of SARS-CoV-2-induced immune dysregulation and VZV latency disruption.^15^^,^^16^ COVID-19-induced T-cell exhaustion and impaired antiviral surveillance likely facilitate VZV reactivation within sensory ganglia during the post-acute phase.^17^

### Uveitis

Uveitis is typically caused by VZV traveling via axonal transport along the trigeminal nerve to intraocular tissues, where local viral replication and cytopathic effects precipitate inflammation. This is supported by VZV DNA and antigens found in the aqueous humor, which correlate with iris atrophy.^18^^,^^19^ The identification of diabetes mellitus, hypertension and smoking as additional risk modifiers in our study is consistent with prior literature linking vascular dysfunction to heightened ocular inflammation.^20^ From a clinical perspective, these findings highlight the need for early ophthalmologic evaluation in post-COVID patients presenting with HZ reactivation, even when classical herpetic eye disease is not apparent. Prompt antiviral therapy, combined with corticosteroids, may prevent complications such as posterior synechiae, cystoid macular edema and secondary glaucoma, which are well-recognized consequences of delayed treatment in herpetic uveitis.^21^

### Optic neuritis

The relationship with optic neuritis aligns with the neurotropic behavior of VZV, which can invade the CNS and cause demyelinating optic neuropathy via direct axonal infection or secondary immune-mediated inflammation.^22^ SARS-CoV-2 may potentiate this risk by inducing persistent immune activation and vascular endothelial injury.^23^^,^^24^ Furthermore, hypertension and alcohol use emerged as additional risk modifiers, consistent with systemic vascular dysregulation contributing to optic nerve vulnerability.^25^ These findings emphasize the importance of maintaining a high index of suspicion for optic neuritis in post-COVID patients presenting with HZ reactivation, particularly given its potential to cause irreversible visual loss if not treated promptly.^26^ Current evidence supports early initiation of antiviral therapy and high-dose corticosteroids for VZV-associated optic neuritis to improve visual prognosis and prevent long-term neuro-ophthalmic morbidity.^27^

### Sensitivity analysis

The primary findings remained highly valid across all sensitivity analyses. Adherence to the proportional hazards assumption and consistent results in competing risk models treating mortality as a competing event further support the statistical validity of our models. The landmark analysis confirmed that the elevated instantaneous hazard for uveitis (approximately 3-fold) and optic neuritis (approximately 2-fold) was temporally persistent over 1-, 2-, and 3-year intervals. Additionally, these risks remained consistently high both before and after PSM. Furthermore, time-window-based analysis confirmed that these instantaneous risks remained consistently high regardless of the specific timing of HZ onset following COVID-19, supporting the stability of the relationship.

To reduce potential misclassification, we verified that outcomes were primarily managed by specialists, with both groups averaging >3 ophthalmology encounters (3.18 vs. 3.02) and >1 ICD-10 entry per patient. This consistency in clinical monitoring and coding across multiple encounters supports high diagnostic ascertainment and minimizes the risk of isolated recording errors.

Furthermore, we observed that patients in the COVID+HZ group exhibited significantly higher hospitalization burdens and progressively increasing COVID-19 reinfection counts over three years. These findings suggest that HZ reactivation often occurs in individuals with greater overall disease severity or immune vulnerability following COVID-19.^28^ The fact that substantial hazards for uveitis and optic neuritis persisted in this population underscores the clinical importance of monitoring for VZV-related complications among those with more severe post-COVID clinical courses.

Crucially, excluding individuals with prior immunosuppression or HIV infection had minimal impact on the results.^29^^,^^30^ This consistency is explained by VZV’s pathophysiology; the neurotropic virus reactivates from latency in sensory ganglia during immune perturbation, causing ocular complications through direct neural invasion or vasculitis.^31^ This sustained relationship underscores the findings’ relevance to immunocompetent individuals and supports a symptom-triggered ophthalmic surveillance approach rather than universal screening in COVID-19 survivors who develop HZ.^32^

### Vaccination and antiviral effects

Exploratory analyses showed COVID-19 vaccination modestly reduced the risk of uveitis (HR = 0.709) and optic neuritis (HR = 0.878) among patients without HZ, but conferred no significant protective effect in the HZ-exposed group. Furthermore, individuals with both COVID-19 and HZ maintained consistently higher risks compared to those without HZ, regardless of vaccination status (vaccinated HRs remained approximately 2.669 and 2.906). Similarly, the receipt of anti-SARS-CoV-2 medications did not significantly modify the 3-year hazard of complications. This highlights that while vaccination may reduce general post-COVID inflammation risk, it does not fully mitigate the elevated neuro-ophthalmic risk associated with VZV reactivation. There is a clear biological rationale for this observation, as VZV is known for its neurotropism and vasculopathic effects, which can lead to inflammation of the uveal tract and optic nerve.^33^ Importantly, COVID-19 vaccines have demonstrated a favorable safety profile, with ocular inflammatory events such as uveitis and optic neuritis reported at very low incidence rates in population-based studies.^34^ These findings support continued vaccination efforts and argue against hesitancy driven by concerns over rare ocular events. However, in individuals who do develop HZ after COVID-19, the risk of serious ocular complications remains significantly elevated, reinforcing the need for targeted ophthalmic monitoring and timely intervention.

### Limitations

This study has several limitations. First, while PSM was employed, residual confounding from unmeasured factors—such as socioeconomic status, lifestyle, and precise vaccination timing or corticosteroid dosing—cannot be fully eliminated. To address this, we confirmed the stability of our findings using an expanded validation set of 29 covariates and by excluding individuals with HIV or prior immunosuppression. Second, although PSM balances baseline states, it cannot fully account for the time-dependent nature of post-COVID events. However, consistent results across landmark, time-window and competing risk analyses alongside supplementary analyses on COVID-19 reinfection and antiviral treatment suggest that these temporal biases did not fundamentally alter our conclusions. Thirdly, while ICD-10 codes carry a risk of misclassification, the comparable frequency of specialist encounters and recurrent coding patterns between groups supports reasonable diagnostic ascertainment and helps mitigate detection bias. Finally, the use of the TriNetX network may restrict generalizability to uninsured or non-US populations. Despite these constraints, the high degree of consistency across diverse analytic approaches underscores the reliability of our findings.

## Conclusion

This large-scale, longitudinal analysis demonstrates that HZ reactivation following COVID-19 is a significant risk factor for subsequent neuro-ophthalmic sequelae: uveitis and optic neuritis. The sustained relationship remains consistent even among immunocompetent individuals and is not fully mitigated by COVID-19 vaccination or systemic antiviral treatments. Given the biologically plausible coherence between SARS-CoV-2-induced immune dysregulation and VZV reactivation, HZ should be clinically recognized as a critical marker of neuro-immune injury. These results support the consideration of symptom-triggered ophthalmic surveillance, although prospective studies are required to confirm these associations and the underlying mechanisms.

## Supplementary Material

hcaf332_Supplementary_Data

## Data Availability

Due to licensing and privacy restrictions, the de-identified, aggregate-level data used in this study from the TriNetX Global Health Research Network are not publicly available. TriNetX provides access to data sourced from a global network of healthcare organizations. Researchers may request access through the TriNetX website (https://trinetx.com) or by contacting Privacy@TriNetX.com. Data are also available from the corresponding author upon reasonable request.
