# Supplementary material for: Increased risk of uveitis and optic neuritis after herpes zoster reactivation in COVID-19: a TriNetX database study
Source: QJM. 2026 Jan 6;119(6):481–90. doi: 10.1093/qjmed/hcaf332 (PMC13391088; doi:10.1093/qjmed/hcaf332)
Supplement: hcaf332_Supplementary_Data [file hcaf332_supplementary_data.docx]

**Section A. Supplementary Tables**

**Supplementary Table S1. Unadjusted Three-Year Risk of Uveitis and Optic Neuritis Following HZ Reactivation: Raw Group Comparison.**

This table provides the crude (unadjusted) Hazard Ratios (HRs) and 95% Confidence Intervals (CIs) for incident uveitis and optic neuritis. The analysis compares the large, unadjusted group of COVID-19 survivors with subsequent HZ reactivation versus the unadjusted group without HZ. These results represent the raw incidence rates prior to Propensity Score Matching (PSM) and should be interpreted alongside the adjusted results presented in Supplementary Table 4.

| **Outcome** | **Group** | **Patients in group** | **Patients with outcome** | **risk** | **Odds ratio**  **(95% CI)** | **Risk difference p-value** |
| --- | --- | --- | --- | --- | --- | --- |
| **Uveitis** | **COVID+HZ** | **109,174** | **605** | **0.006** | **3.934**  **(3.297–4.694)** | **0.000** |
|  | **COVID–HZ** | **109,580** | **155** | **0.001** |  |  |
| **Optic neuritis** | **COVID+HZ** | **109,647** | **211** | **0.002** | **2.679**  **(2.069–3.470)** | **0.000** |
|  | **COVID–HZ** | **109,853** | **79** | **0.001** |  |  |

**Abbreviations:** COVID+HZ: COVID-19 patients with herpes zoster reactivation; COVID-HZ: COVID-19 patients without herpes zoster reactivation; CI: confidence interval

**Supplementary Table S2. Sensitivity Analysis: Comparative Three-Year Risk of Uveitis and Optic Neuritis Following HZ Reactivation in Age-Stratified and Overall Groups**

This table presents the Hazard Ratios (HRs) and 95% Confidence Intervals (CIs) for uveitis and optic neuritis, comparing COVID-19 survivors with HZ reactivation to matched controls without HZ. The analysis is performed across two distinct study populations: the overall adult group (18–89 years) and a predefined age-restricted group (50–59 years). The consistency of the risk estimates across these analyses supports the observed relationship, demonstrating that the increased hazard is not solely attributed to extreme age groups.

**
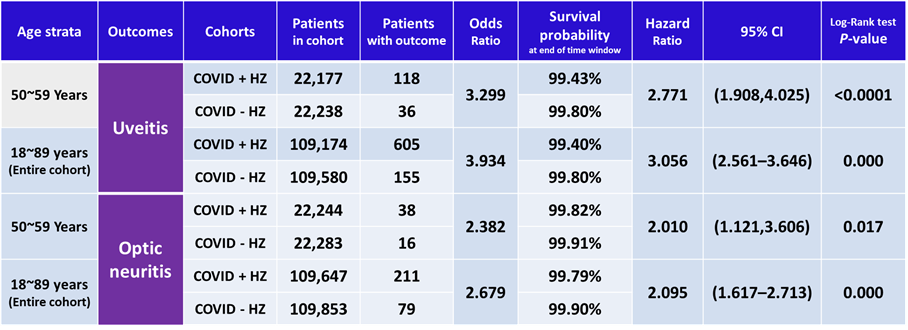
** **Abbreviations:** COVID+HZ: COVID-19 patients with herpes zoster reactivation; COVID-HZ: COVID-19 patients without herpes zoster reactivation; CI: confidence interval

**Supplementary Table S3. Landmark Sensitivity Analysis: Time-Dependent Hazard Ratios for Uveitis and Optic Neuritis Following HZ Reactivation.**

This table presents the results of the landmark analysis, showing the Hazard Ratios (HRs) and 95% Confidence Intervals (CIs) for incident uveitis and optic neuritis at the 1-year, 2-year, and 3-year time points. This methodology was utilized to assess the stability and persistence of the increased neuro-ophthalmic risk associated with HZ reactivation throughout the follow-up period, demonstrating that the heightened risk is sustained over time and not solely confined to the immediate post-HZ period.


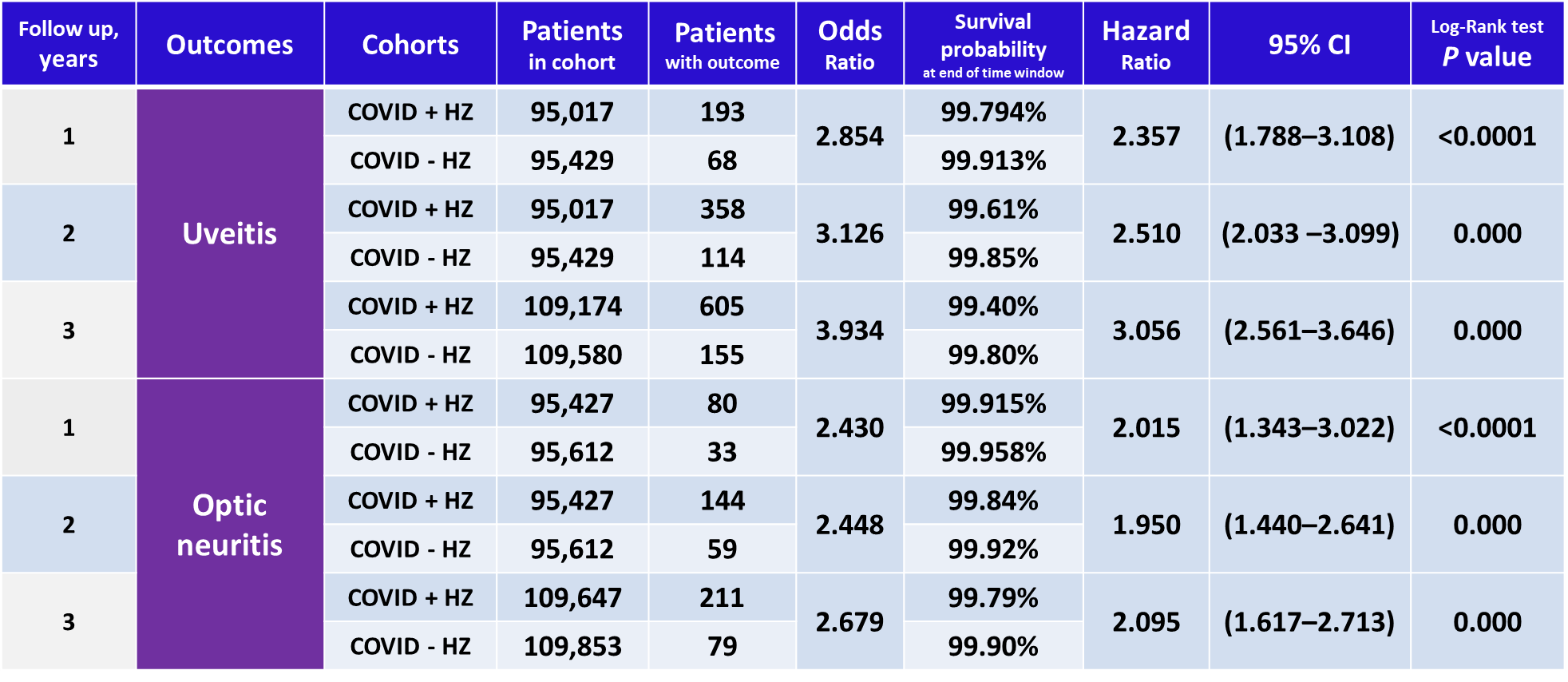


**Abbreviations:** COVID+HZ: COVID-19 patients with herpes zoster reactivation; COVID-HZ: COVID-19 patients without herpes zoster reactivation; CI: confidence interval

**Supplementary Table S4. Comparison of Three-Year Hazard Ratios for Uveitis and Optic Neuritis: Analysis Before and After Propensity Score Matching (PSM).**

This table presents the Hazard Ratios (HRs) and 95% Confidence Intervals (CIs) for incident uveitis and optic neuritis, comparing the HZ-exposed and HZ-unexposed groups. The results are presented for both the unmatched raw data and the 1:1 Propensity Score Matched (PSM) groups. The minimal change in the magnitude and significance of the risk estimates after rigorous PSM confirms the consistent relationship between HZ reactivation and subsequent neuro-ophthalmic sequelae, minimizing the potential impact of measured confounding variables.


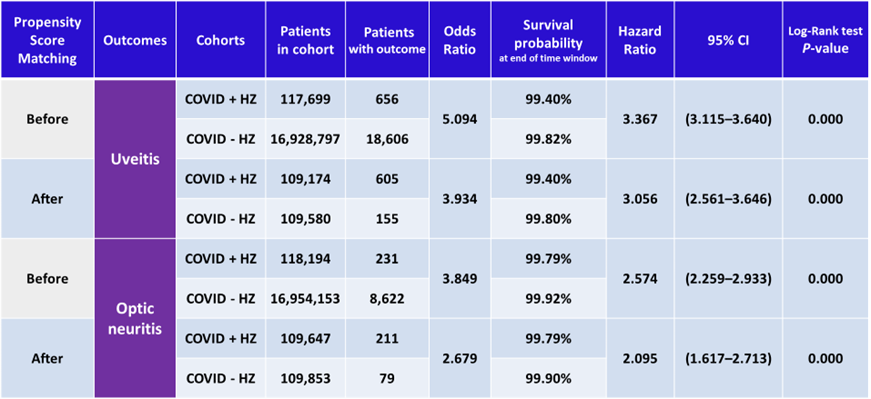


**Abbreviations:** COVID+HZ: COVID-19 patients with herpes zoster reactivation; COVID-HZ: COVID-19 patients without herpes zoster reactivation; CI: confidence interval

**Supplementary Table S5. Sensitivity Analysis: Risk of Uveitis and Optic Neuritis Following HZ Reactivation in the Immunocompetent Group.**

This table presents the Hazard Ratios (HRs) and 95% Confidence Intervals (CIs) for incident uveitis and optic neuritis after HZ reactivation, specifically from the analysis excluding all patients with documented immunosuppression (e.g., specific diseases, active chemotherapy, or use of immunosuppressive medications) within one year prior to the COVID-19 index date. The sustained magnitude and statistical significance of the risk estimates in this immunocompetent population underscore the consistency of the relationship and demonstrate that the increased hazard is not exclusively accounted for by pre-existing immune compromise.


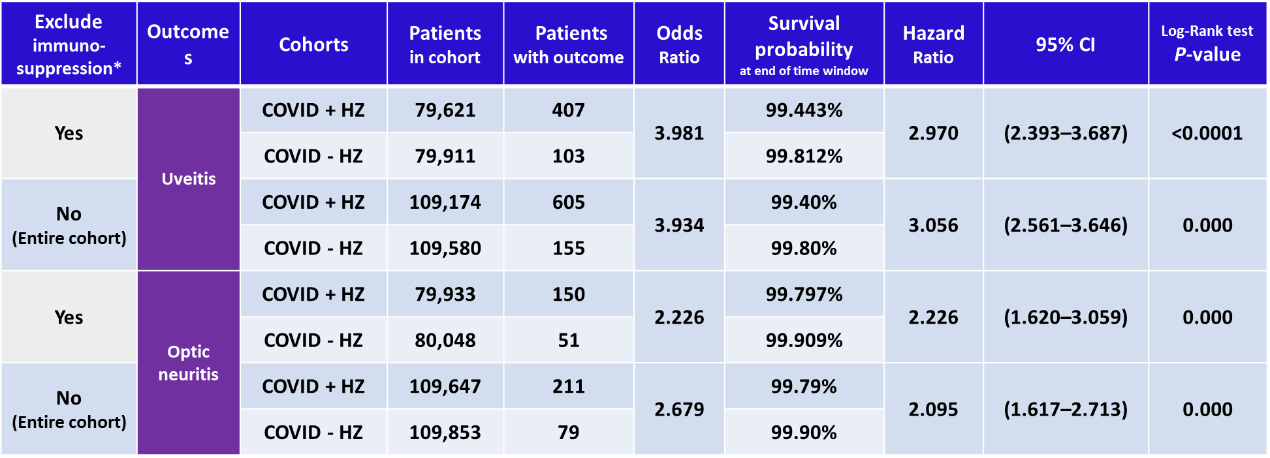


**Abbreviations:** COVID+HZ: COVID-19 patients with herpes zoster reactivation; COVID-HZ: COVID-19 patients without herpes zoster reactivation; CI: confidence interval;

**Supplementary Table S6. Sensitivity Analysis: Three-Year Risk of Uveitis Following HZ Reactivation Stratified by Receipt of Anti–SARS-CoV-2 Medications.**

This table presents the results of a sensitivity analysis assessing the potential confounding effect of anti–SARS-CoV-2 medications on the risk of uveitis following herpes zoster (HZ) reactivation. The analysis compares 1,826 patients who received antiviral treatment with 1,824 untreated controls. The results demonstrate that the absolute risk of uveitis was low in both groups (0.712% vs. 0.603%) and did not differ significantly (Risk Difference p = 0.684; Log-Rank p = 0.659). These findings suggest that the observed relationship between HZ reactivation and neuro-ophthalmic complications remains consistent regardless of COVID-19 antiviral therapy.

**
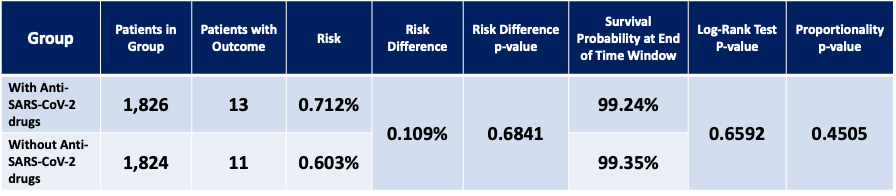
**

**Footnotes:**
p-value (Risk Difference): Calculated using the Chi-square test for absolute risk at the end of the follow-up period.

p-value (Log-Rank): Calculated using the Kaplan-Meier method to assess differences in survival distributions.

**Note:** Due to the small number of optic neuritis events in this specific sub-group, stratified analyses for optic neuritis were not feasible.

**Supplementary Table S7A. Comparison of Three-Year Ophthalmology Specialist Encounters and Procedures Between Groups.**

This table compares the intensity of ophthalmology-specific healthcare utilization between the two groups over the three-year follow-up period. The mean number of ophthalmology specialist encounters and procedures serves as a proxy for diagnostic surveillance intensity. While a small statistical difference exists (3.18 vs. 3.02), the proximity of these values suggests that both groups received comparable ophthalmic monitoring, minimizing the risk of detection bias.

**
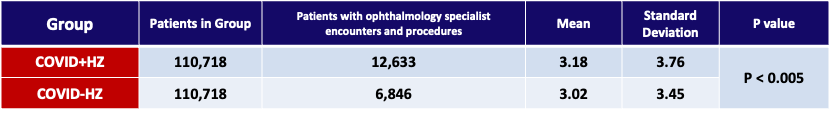
**

**Abbreviations:** COVID+HZ: COVID-19 patients with herpes zoster reactivation; COVID-HZ: COVID-19 patients without herpes zoster reactivation; SD, standard deviation.

**Footnotes:**
Data Source: Metrics derived from the TriNetX global health research network.
Statistical Test: p-value calculated using an independent t-test for comparison of means.

**Supplementary Table S7B. Diagnostic Coding Frequency and Ascertainment for Uveitis and Optic Neuritis.**

This table presents the frequency of ICD-10 diagnostic coding for uveitis and optic neuritis cases. A mean frequency of greater than one code per patient indicates that diagnoses were consistently recorded over multiple clinical encounters rather than as isolated entries. The similarity in coding frequency between the COVID-19+HZ and COVID-19–HZ groups confirms high diagnostic ascertainment and consistency across the study groups.

**
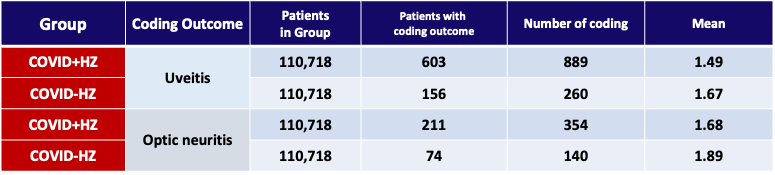
**

**Abbreviations:** COVID+HZ: COVID-19 patients with herpes zoster reactivation; COVID-HZ: COVID-19 patients without herpes zoster reactivation

**Footnotes:**

Diagnostic Criteria: Based on repeated occurrences of specific ICD-10 codes for uveitis and optic neuritis within the electronic health record.

Interpretation: High diagnostic consistency (mean > 1) supports the validity of the study outcomes.

**Supplementary Table S8. Comparison of Three-Year Cumulative Hospitalization Burden Between Groups**

This table compares the cumulative hospitalization burden over a three-year follow-up period between patients who experienced herpes zoster (HZ) reactivation following COVID-19 and matched controls. Patients in the COVID-19+HZ group demonstrated a significantly higher frequency of hospital admissions (Mean 1.851 vs. 1.011; p < 0.001) and a higher absolute number of patients requiring at least one hospitalization (36,224 vs. 26,666). These findings indicate that HZ reactivation is associated with a substantially increased systemic clinical burden beyond ocular complications.


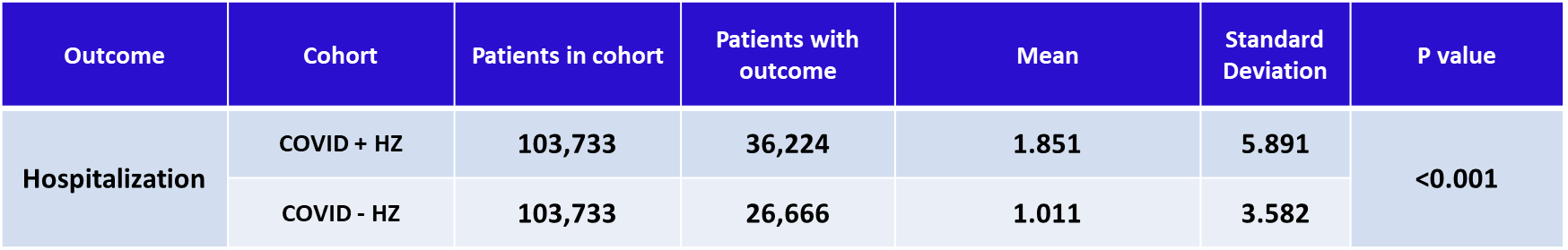


**Abbreviations:** COVID+HZ: COVID-19 patients with herpes zoster reactivation; COVID-HZ: COVID-19 patients without herpes zoster reactivation

**Footnotes:**

- **p-value:** Calculated using an independent t-test to compare the mean hospitalization counts between the two groups.
- **Matching:** Groups were propensity-score matched for baseline demographics and comorbidities to ensure comparability.
- **Abbreviations:** HZ, herpes zoster; SD, standard deviation.

**Supplementary Table S9. Comparison of Cumulative COVID-19 Reinfections Between Groups Over 3 Years.**

This table presents the mean number of COVID-19 reinfections at specific follow-up intervals (6 months, 1 year, 2 years, and 3 years). A t-test was used to compare the cumulative burden of reinfections between the COVID+HZ and COVID-HZ groups.

**
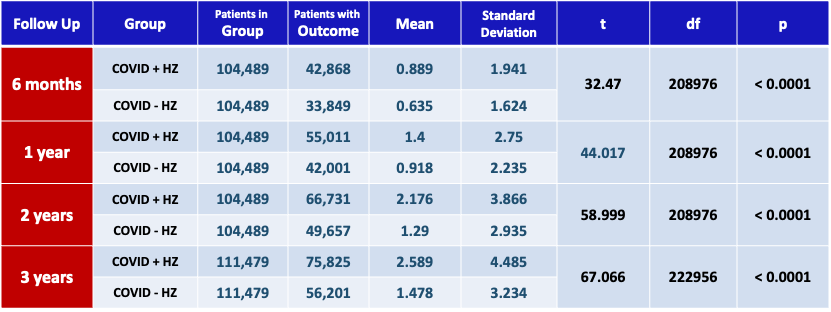
**

**Abbreviations**: COVID+HZ, COVID-19 patients with herpes zoster reactivation; COVID-HZ, COVID-19 patients without herpes zoster reactivation; df, degrees of freedom; SD, standard deviation.

**Note:** A t-test was performed to compare the mean number of cumulative COVID-19 reinfections between the two groups at each follow-up interval. Statistical significance was defined as p < 0.05.

**Supplementary Table S10. Time-Window–Based Sensitivity Analysis Stratified by the Timing of Herpes Zoster Onset.**

To address the potential influence of time-varying factors, this analysis utilizes a time-window approach, stratifying the study groups based on the interval between COVID-19 diagnosis and the onset of herpes zoster (within 6, 12, 24, and 36 months).

**
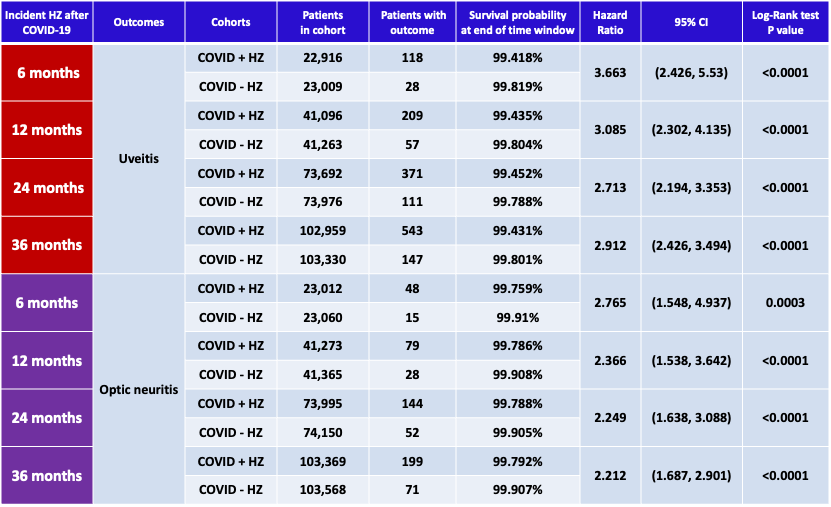
**

**Abbreviations:** CI, confidence interval; COVID+HZ, COVID-19 patients with herpes zoster reactivation; COVID-HZ, COVID-19 patients without herpes zoster reactivation; HR, hazard ratio.

**Note:** The TriNetX platform is a real-world, dynamically updated database; this table was generated on December 14, 2025. This time-window–based sensitivity analysis evaluates the risks of uveitis and optic neuritis according to the specific timing of HZ onset following COVID-19. $p$-values were derived from log-rank tests.

**Supplementary Table S11. Assessment of Proportional Hazards Assumption for Ocular Inflammatory Outcomes.**

This table evaluates the proportional hazards (PH) assumption using the Grambsch–Therneau test. The non-significant p-values for both uveitis and optic neuritis confirm that the hazard ratios remained constant over the 3-year follow-up period, validating the use of Cox proportional hazards modeling.


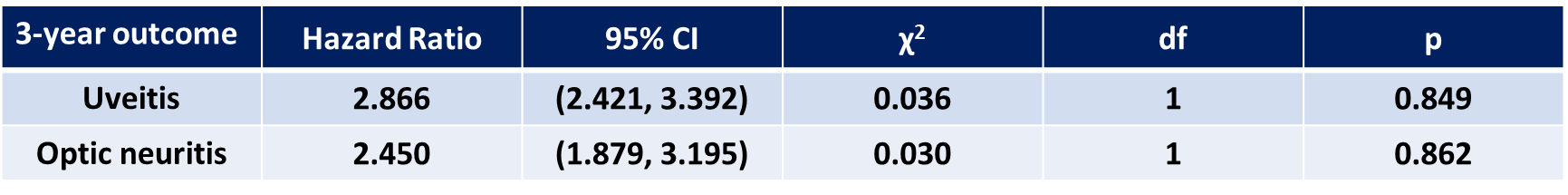


**Abbreviations:** CI, confidence interval; COVID+HZ, COVID-19 patients with herpes zoster reactivation; COVID-HZ, COVID-19 patients without herpes zoster reactivation; HR, hazard ratio; PH, proportional hazards.

**Note:** The PH assumption was tested using the Grambsch–Therneau method based on scaled Schoenfeld residuals. A p-value $> 0.05 indicates that the hazard ratios are stable over time and the proportionality assumption holds.

**Supplementary Table S12. Competing Risk Analysis of Ocular Inflammatory Outcomes with Mortality as a Competing Event.**

This table presents the cumulative incidence of ocular inflammatory outcomes (uveitis and optic neuritis) alongside all-cause mortality over a 3-year follow-up period. To address potential survival bias, a competing risk analysis was performed using the Aalen–Johansen estimator, where mortality was treated as a competing event. This ensures a robust estimation of the incidence of ocular complications in the presence of death as a competing risk.

**
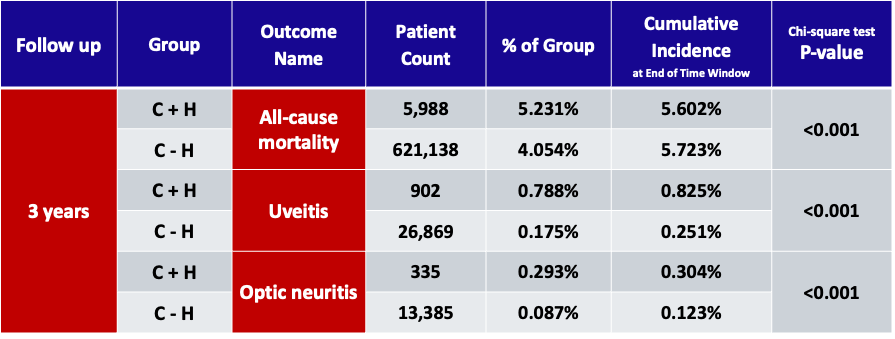
**

**Abbreviations: C**+H, COVID-19 patients with herpes zoster reactivation; C-H, COVID-19 patients without herpes zoster reactivation.

**Note:** Cumulative incidence was calculated using the Aalen–Johansen estimator to account for mortality as a competing risk. p-values were derived from Chi-square tests comparing the frequency of events between the two groups. The total population sizes at the 3-year mark were approximately 114,000 for the C+H group and 15.3 million for the C-H group.

**Section B. Supplementary Figures**

**Supplementary Figure S1. Love Plot Illustrating Covariate Balance for the 50–59 Years Age Subgroup.**


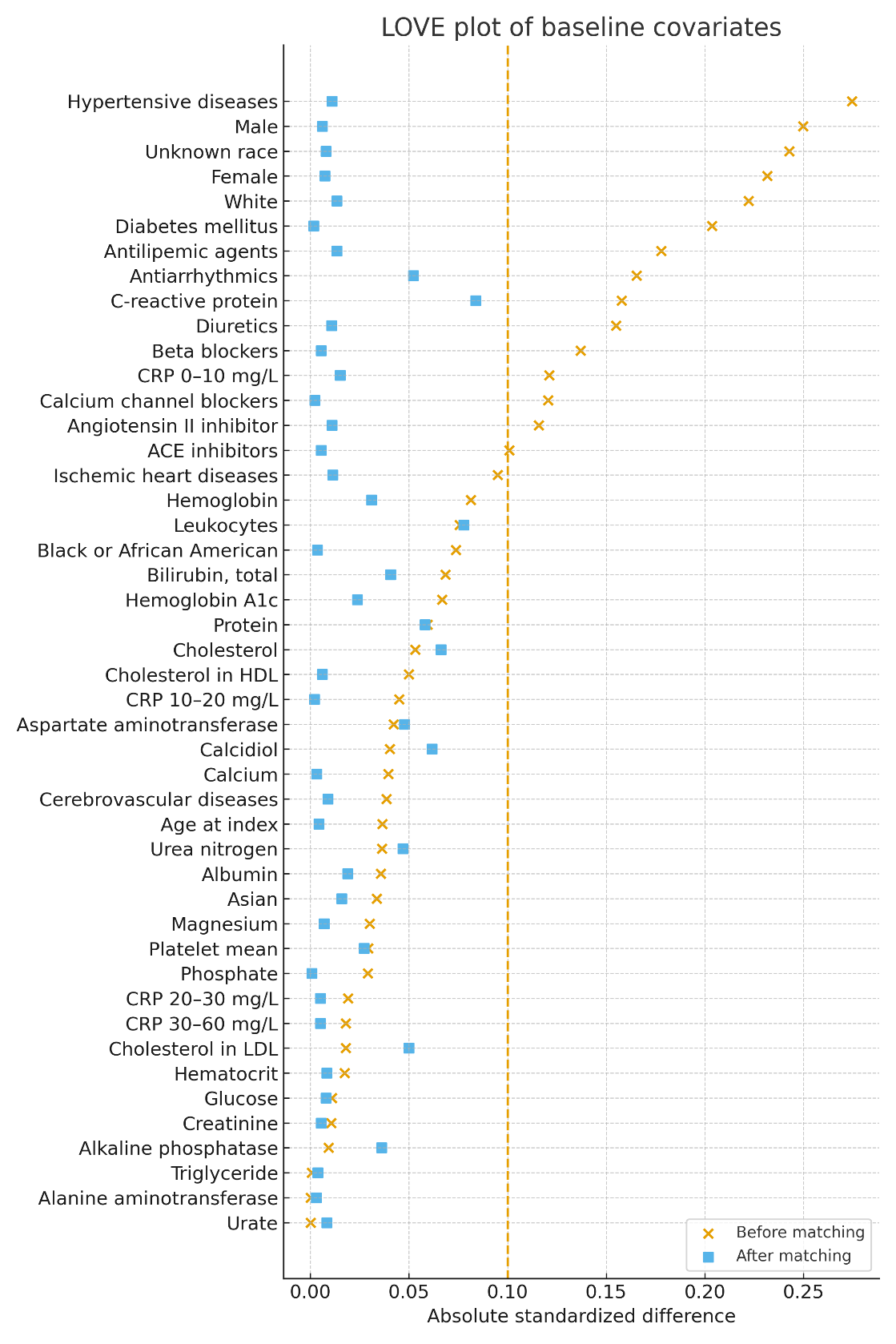


**Legend:**

This Love plot displays the absolute standardized mean differences (ASMD) for all baseline covariates between the COVID-19+HZ and COVID-19–HZ groups within the 50–59 years age stratum.

- Y-axis: Represents all included baseline variables, including demographics, clinical diagnoses, laboratory values (including CRP categories), and medication history.
- X-axis: Represents the absolute standardized mean difference.
- Threshold: The dashed vertical line at 0.1 marks the standardized threshold for group balance.

**Interpretation:** Before matching (indicated by open circles), several variables exceeded the 0.1 threshold, suggesting potential selection bias. After 1:1 propensity score matching (indicated by solid dots), all covariates achieved an ASMD of less than 0.1, indicating that a high degree of balance was achieved across all clinical and laboratory parameters.

**Abbreviations:** ASMD, absolute standardized mean difference; COVID+HZ, COVID-19 patients with herpes zoster reactivation; COVID-HZ, COVID-19 patients without herpes zoster reactivation; CRP, C-reactive protein; HZ, herpes zoster.

**Supplementary Figure S2. Longitudinal Trend of Cumulative COVID-19 Reinfections Over 3 Years.**

**Legend:** This figure illustrates the mean cumulative number of COVID-19 reinfections in the COVID+HZ group (red line) and the COVID-HZ group (blue line) over the 3-year follow-up period. The error bars represent the 95% confidence intervals. As shown, the burden of reinfection was significantly higher in patients with post-COVID herpes zoster reactivation at all time points (p < 0.0001).


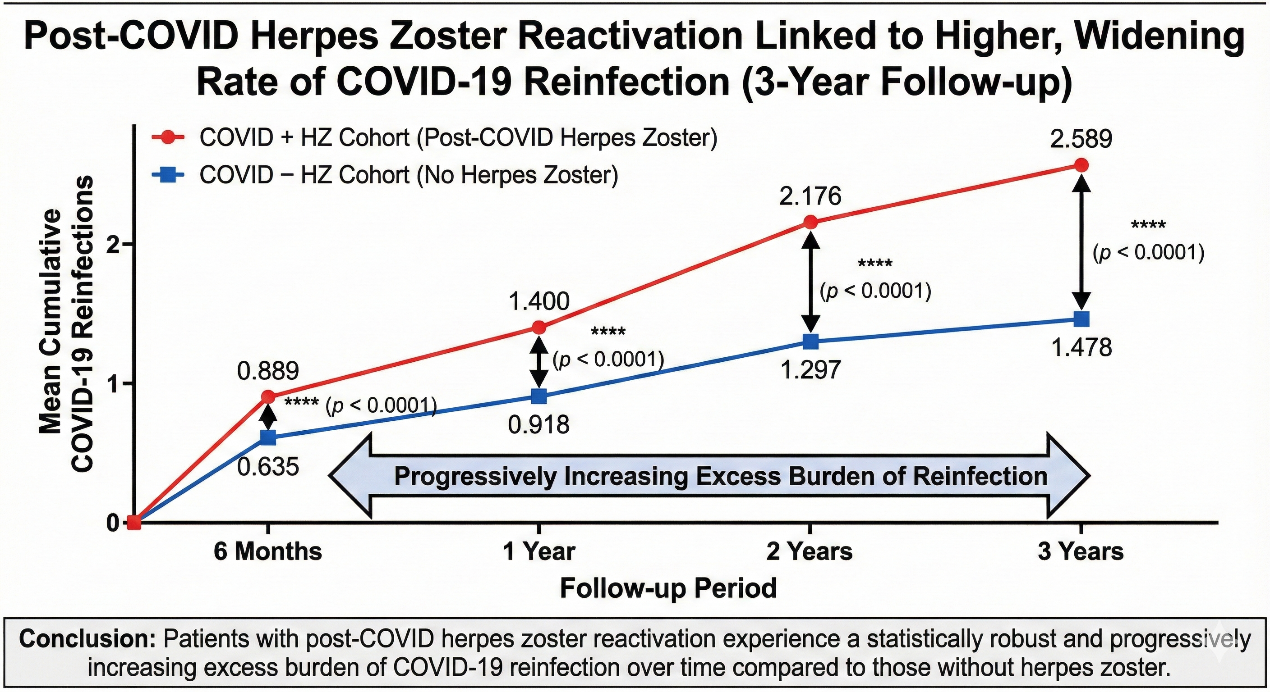


**Abbreviations:** COVID+HZ, COVID-19 patients with herpes zoster reactivation; COVID-HZ, COVID-19 patients without herpes zoster reactivation.

**Supplementary Figure S3.** **Risk of Ocular Inflammation and Hospitalization Post-COVID-19 with Herpes Zoster as a Time-Varying Covariate.**

**Legend:** This figure illustrates the hazard ratios (HRs) and associated risks for ocular inflammation and hospitalization, treating herpes zoster (HZ) reactivation as a time-varying covariate. (A) Cumulative hazard of ocular inflammatory outcomes following COVID-19 infection. (B) Cumulative risk of all-cause hospitalization. Even when accounting for the variable timing of HZ onset, the COVID+HZ group consistently exhibits a significantly higher risk compared to the COVID-HZ group ($p < 0.05$). This analysis supports the consistency of the relationship between HZ and adverse outcome, suggesting that the findings are not significantly influenced by the timing of exposure onset.


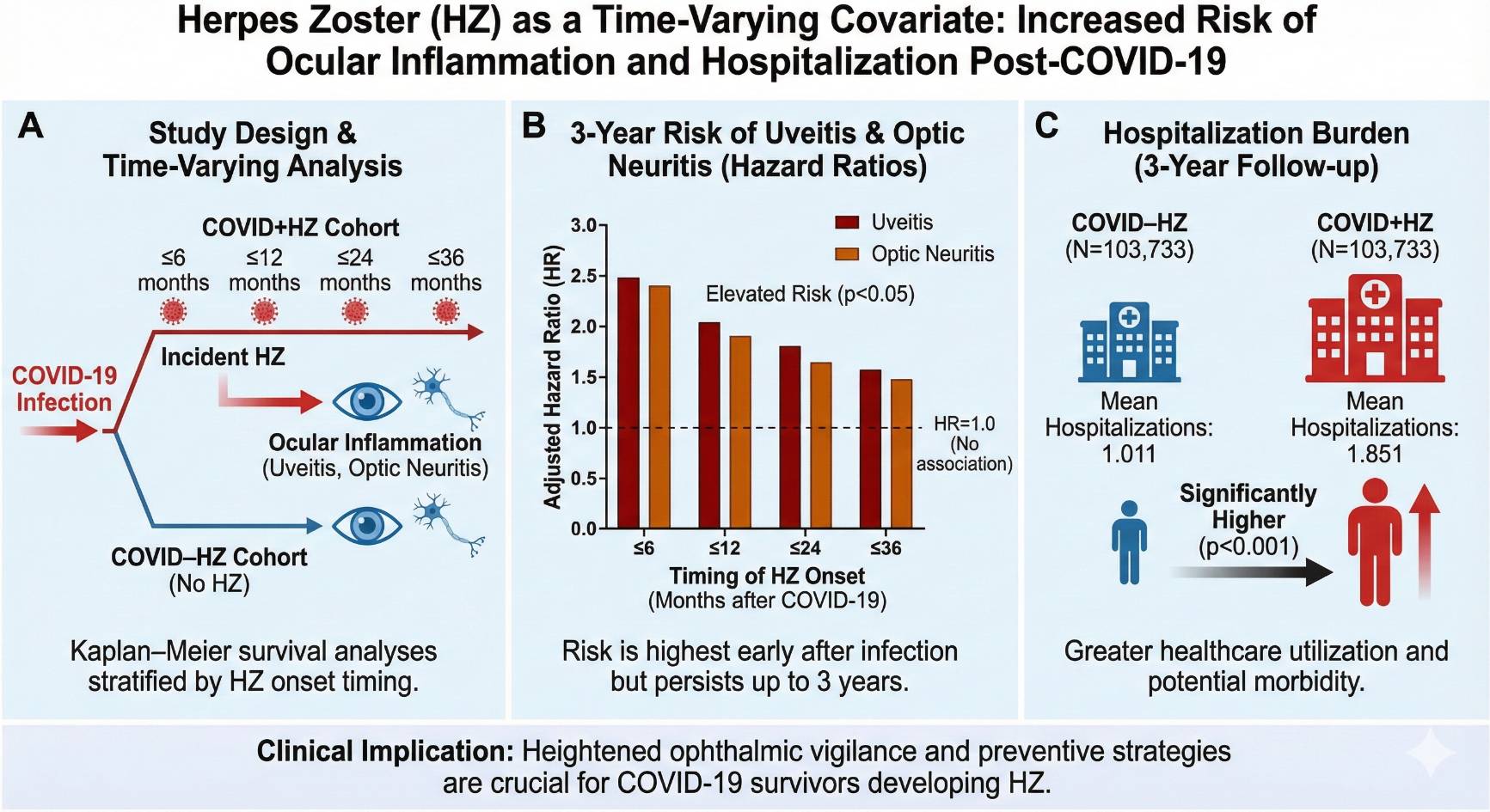


**Abbreviations:** COVID+HZ, COVID-19 patients with herpes zoster reactivation; COVID-HZ, COVID-19 patients without herpes zoster reactivation; HR, hazard ratio.

**Supplementary Figure S4. Cumulative Incidence Curves for Ocular Inflammatory Outcomes Accounting for Competing Risk of Death.**

**Legend:** Cumulative incidence curves for uveitis and optic neuritis were generated using the Aalen–Johansen estimator. Mortality during the follow-up period was accounted for as a competing risk.


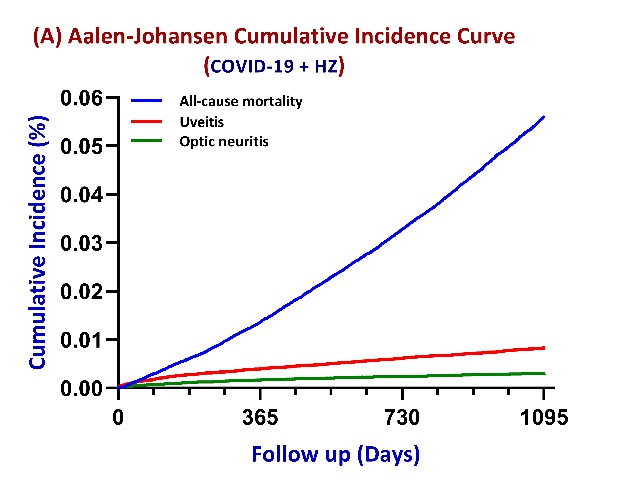

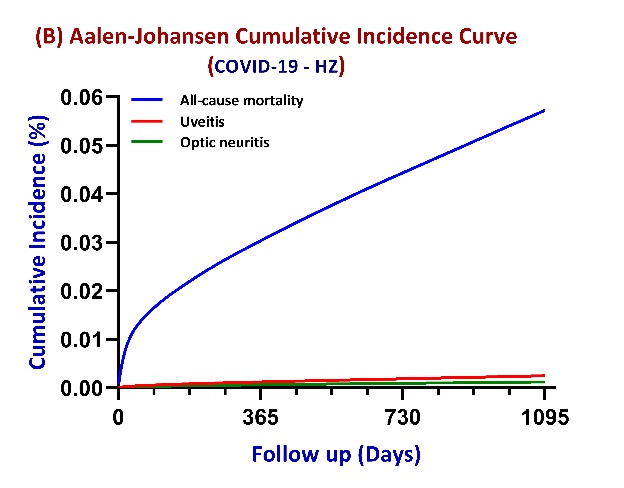


**Abbreviations:** COVID-19+HZ, COVID-19 patients with herpes zoster reactivation; COVID-19-HZ, COVID-19 patients without herpes zoster reactivation.
